# Fabrication and Optimisation of Alumina Nanoporous Membranes for Drug Delivery Applications: A Comparative Study

**DOI:** 10.3390/nano14131078

**Published:** 2024-06-24

**Authors:** Lamyaa Osama, Hala T. Handal, Sara A. M. El-Sayed, Emad M. Elzayat, Mostafa Mabrouk

**Affiliations:** 1 Refractories, Ceramics and Building Materials Department, National Research Center, 33El Bohouth St. (Former EL Tahrir St.), Dokki, Giza P.O. Box 12622, Egypt; lamyaa.osama.moh@gmail.com (L.O.); sarali_87@yahoo.com (S.A.M.E.-S.); 2 Inorganic Chemistry Department, National Research Center, Cairo P.O. Box 12622, Egypt; hally.handal@gmail.com; 3 Biotechnology Department, Faculty of Science, Cairo University, Giza P.O. Box 12613, Egypt; elzayat.emad@yahoo.com; 4 Academy of Scientific Research and Technology (ASRT), Cairo P.O. Box 11516, Egypt

**Keywords:** neurodegenerative disorders, implantable nanoporous membranes, AAO, SEM, AFM, kinetic studies

## Abstract

Neurodegenerative disorders cause most physical and mental disabilities, and therefore require effective treatment. The blood–brain barrier (BBB) prevents drug molecules from crossing from the blood to the brain, making brain drug delivery difficult. Implantable devices could provide sustained and regulated medication to solve this problem. Two electrolytes (0.3 M oxalic acid and 0.3 M sulphuric acid) were used to anodise Al_2_O_3_ nanoporous membranes, followed by a third anodisation in concentrated H_2_SO_4_ to separate the through-hole membranes from the aluminium substrate. FTIR, AFM, and SEM/EDX were used to characterise the membranes’ structure and morphology. The effects of the anodisation time and electrolyte type on the AAO layer pore density, diameter, interpore distance, and thickness were examined. As a model drug for neurodegenerative disorders, donepezil hydrochloride (DHC) was loaded onto thin alumina nanoporous membranes. The DHC release profiles were characterised at two concentrations using a UV–Vis spectrophotometer. Oxalic acid membranes demonstrated an average pore diameter of 39.6–32.5 nm, which was two times larger than sulphuric acid membranes (22.6–19.7 nm). After increasing the anodisation time from 3 to 5 h, all of the membranes showed a reduction in pore diameter that was stable regardless of the electrolyte type or period. Drug release from oxalic acid-fabricated membranes was controlled and sustained for over 2 weeks. Thus, nanoporous membranes as implantable drug delivery systems could improve neurodegenerative disease treatment.

## 1. Introduction

Neurons are essential for proper brain function because they play a critical role in communication. The loss of their structure and/or function causes neurodegeneration, which is considered the main reason for the pathogenesis of several brain diseases [1]. Damage to synapses, problems with neural networks, and the build-up of proteins with changed chemical properties are all signs of neurodegeneration (ND) [2,3,4,5]. About 15% of the world’s population suffers from neurological disorders, which are considered the primary cause of physical and mental disability [6]. The most prevalent NDs are spinal muscular atrophy, Parkinson’s disease, Alzheimer’s disease, amyotrophic lateral sclerosis, motor neuron disease, and spinocerebellar ataxia [7,8,9,10].

In Alzheimer’s disease (AD), harmful protein clusters like β-amyloid proteins in the form of plaques and hyperphosphorylated tau proteins in the form of tangles are present [11]. Some examples of cholinesterase inhibitors (AChEIs) are galantamine, donepezil, and rivastigmine. They stop acetylcholine (ACh) from breaking down, which makes cholinergic neurotransmission better. ACh is considered a crucial neurotransmitter, as it interacts with memory and learning receptors in the nervous system. Currently, we can use all widely prescribed AChEIs to treat mild-to-moderate stages of AD, but only donepezil can treat all stages (mild, moderate, and severe). Additionally, donepezil has fewer interactions with other medications, has a longer half-life, is easier to tolerate because it targets central AChEIs more specifically, and food does not seem to change the medication’s pharmacokinetics [12].

The blood–brain barrier (BBB) is known to be a diffusion barrier that inhibits the blood-circulating substances from reaching the brain, thus maintaining homeostasis and allowing normal brain functioning [13]. The drawbacks of existing therapeutics and the BBB’s limitations have led to a great need for novel therapeutic strategies for ND treatments [14]. Nanotechnology is a branch of science that creates materials for targeted drug delivery to the central nervous system (CNS) [15,16]. This technology utilises nanoscale materials, with sizes ranging from 1 to 1000 nm, which have the ability to interact with biological systems at the molecular level [17]. Researchers have fabricated nanoparticles from a variety of compounds, such as inorganic minerals and synthetic and natural polymers. One of the most significant templates for the creation of nanostructures are nanoporous membranes, which can also act as carriers for nanoparticles. Drug delivery by nanocarriers to the brain has proven to be quite effective [18], and ordered porous surfaces can serve as reservoirs for drugs by using different techniques to fill in the pores. Nanocarriers have many great qualities, making them an interesting way to treat NDs. These include being able to hold a lot of drugs, not being too harmful to the body as a whole, letting more drugs through, and being very stable chemically and physically [1]. Therefore, we can control the porosity of the membranes to modify the rate of pharmaceutical release [19]. Precursors used for the fabrication of nanoporous membranes could be organic, inorganic, or composite materials. Silicon-based products and anodised aluminium oxide (AAO) are some examples of inorganic compounds [20,21].

AAO membranes possess a well-organised, homogeneous structure with precise control over the dimensions and arrangement of their features. The electrochemical anodisation process allows for the facile and cost-effective production of ordered hexagonal porous structures, making it an attractive choice for large-scale manufacturing compared to more complex techniques [22]. The specific parameters of the anodisation process, such as time, temperature, voltage, and electrolyte type and concentration (e.g., phosphoric, sulphuric, or oxalic acids), can be tuned to modulate the distribution, shape, and size of the structural elements (e.g., the depth, barrier thickness, diameter, and spacing) [19].

Despite these advantages, researchers face challenges during the fabrication and separation of AAO membranes. Achieving the desired structural profile requires the careful optimisation of numerous factors, including the applied voltage, electrolyte composition, anodisation sequence, and duration [21,23]. Moreover, the separation methods commonly employed are often sophisticated, irreproducible, and time-consuming, with some techniques even causing pore wall dissolution and structural damage.

Furthermore, the inherent low mechanical strength of AAO membranes can lead to the production of fragmented samples. To address these challenges, this study aimed to optimise the conditions for the preparation of stable AAO membranes with controlled structural features using the electrochemical anodisation technique. Various parameters, including the anodisation time and electrolyte type, were investigated to precisely modulate the pore profile (e.g., the diameter, spacing, density, and length) and chemistry (e.g., the crystallographic phase, distribution, and impurity content). This level of control is crucial for producing highly defined nanostructured AAO templates that can be used to manipulate the drug volume and regulate its release. As a model application, the optimised membranes were utilised for the in vitro loading and release of donepezil hydrochloride, a drug for the treatment of Alzheimer’s disease, in artificial cerebrospinal fluid.

## 2. Materials and Methods

### 2.1. Materials

Aluminium foil (Al, 99.998%, 0.5 mm thick, Alfa Aesar, Ward Hill, MA, USA), acetone (C_3_H_6_O, 99.3%, Piochem, Giza, Egypt), sodium hydroxide (NaOH, 39.997 g/mol, El-Nasr pharmaceutical chemicals company, Cairo, Egypt), absolute ethanol (CH_3_CH_2_OH, 46.07 g/mol, Merck, Darmstadt, Germany), perchloric acid (HClO_4_, 71–73%, Advent, Mumbai, India), oxalic acid dihydrate (H_2_C_2_O_4_·2H_2_O, 126.07 g/mol, Advent, Mumbai, India), chromium trioxide extra pure (CrO_3_, 99.99 g/mol, Laboratory Rasayan, Gujarat, India), orthophosphoric acid (H_3_PO_4_, 85%, El Nasr pharmaceutical chemicals company, Cairo, Egypt), sulphuric acid (H_2_SO_4_, 95–97%, Merck, Darmstadt, Germany), copper (II) chloride (CuCl_2_, 134.45 g/mol, Advent, Mumbai, India), hydrochloric acid (HCl, 37%, El Nasr pharmaceutical chemicals company, Cairo, Egypt), and donepezil hydrochloride (C_24_H_30_ClNO_3_, ≥98% (HPLC), Sigma Aldrich, Lu Wan Qu, Shanghai, China).

### 2.2. Experiments

#### 2.2.1. Preparation of Alumina Nanoporous Membranes

Nanoporous alumina membranes were prepared using a two-step anodisation process at 0 °C. Before anodisation, a high-purity, 0.5 mm thick Al foil was heat-treated in air at 500 °C for 3 h. Then, surface treatment for the foil was performed in acetone for 10 min to remove the grease and in 1 M sodium hydroxide for 3 min to dissolve the Al_2_O_3_. Subsequently, it was thoroughly rinsed with Milli-Q water. After that, the Al foil was electrochemically polished in a mixture of perchloric acid and ethanol in a 1:3 (V/V) ratio, resulting in a mirror-like film. In the electrochemical system, the Al sheet acts as an anode that faces a platinum electrode cathode. The electropolishing voltage was adjusted to 20 V for 3 min in an ice bath.

**Figure 1:**
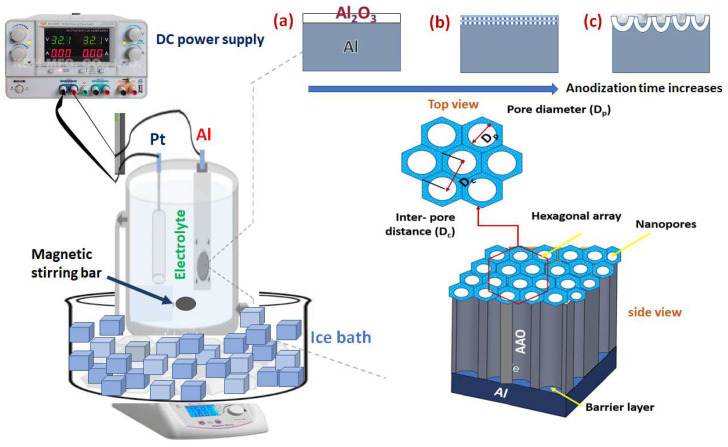
Schematic diagram of a typical anodising setup showing both the top and cross-section views of a fully grown nano-tube array (**a**) thin layer of oxide (barrier layer) forms at the early beginning of the anodization, (**b**) small pits to form porous layer on the formed Al_2_O_3_ membrane, and (**c**) nano-pore growth in a honeycomb structure.

The Al_2_O_3_ nanoporous membrane was fabricated through two anodisation processes in a homemade anodisation cell (shown in Figure 1), as described elsewhere [23]. To obtain nanoporous AAO structures, the anodisation cell contained 50 mL of 0.3 M oxalic acid (or 0.3 M sulphuric acid) at a potential of approximately 40 V (or at 25 V for the sulphuric acid) for 5 h. Anodisation was carried out at 0 °C to remove the generated Joule heat and induce the rapid growth of anodic oxides without further burning. The second anodisation step was carried out similarly to the first anodisation step but for different periods (3, 5, 7, and 10 h). Between the two-step anodisation, a selective removal or stripping of the porous oxide layer formed in the first anodisation step was performed in a mixed solution of 1.8 wt% chromium trioxide and 6 V% orthophosphoric acid solutions at 70 °C to pre-structure the aluminium (Al) surface (textured surface). This step was required to ensure the self-organisation of flawless pores from up to down the tube during the second anodisation step. The resulting inner surface of the Al displayed a uniform concave nano-array, providing a foundation for building an ordered pore size distribution. According to [24], porous alumina has a double structure, which is made up of a barrier and porous layers. The porous alumina layer was formed with hexagonal cells that had nanopores in their centres. At the bottom of the pores, a continuous, dielectric oxide layer was formed, which is called the barrier layer (Figure 1).

#### 2.2.2. Different Separation Techniques of AAO Nanoporous Membranes

To separate the AAO membrane from the underlying Al sheet, different procedures were performed to fabricate free-standing and through-hole AAO arrays.

##### Chemical Method

In this method, the AAO/Al was first put in a copper (II) chloride and hydrochloric acid solution to remove the Al. Then, it was immersed in 5 wt.% orthophosphoric acid to remove the barrier layer and open the pores at the bottom of the membrane. A protective layer made of clay was coated on the top surface of the AAO/Al sheet to give mechanical strength to the membrane and to prevent the over-etching of the surface structure and the uneven diffusion of the H_3_PO_4_ acid into the nanopores (seen in Figure S1) during the removal of the barrier layer. Then, we selectively etched away the unreacted aluminium substrate by immersing it in a solution containing 3.5 g copper (II) chloride and 100 mL hydrochloric acid (37%) [25].

##### Voltage Pulse Detachment Method

In this technique, we performed electrolysis at a high voltage, as previously reported by Yuan et al. [26]. The AAO was separated as follows: after the second anodisation step, a stepwise voltage reduction in the oxalic acid from the formation voltage (i.e., 40 V) to 10 V was performed. This consequently led to a decrease in the barrier layer’s thickness. A voltage-drop process was applied to partially penetrate the barrier layer. Anodic polarisation in a mixed solution of perchloric acid and ethanol (1:1 wt.%) for 3 sec was repeated several times at a voltage of 50 V, which was higher than the formation voltage that was applied. The detached anodic films, i.e., the AAO membranes, were chemically etched in 5 wt.% phosphoric acid at 30 °C to remove any residual barrier layers. It was expected that the intensified electric field-assisted dissolution of the oxide layers would be enhanced by the chemical dissolution induced by Joule heating [26].

##### The Wet Etching of Two-Layered Anodic Porous Alumina

In the third technique, the AAO/Al was anodised for a third time in 12 M H_2_SO_4_ at a constant voltage similar to the formation voltage, i.e., either 40 V or 25 V, at 0 °C for 20 min (for every 90 min of the second anodisation step). The detachment of the membrane was carried out in the mixed solution of CrO_3_ and H_3_PO_4_ at 30 °C for 15 min (for every 90 min of the second anodisation step) [27,28,29].

### 2.3. Characterisation of AAO Membranes

#### 2.3.1. Scanning Electron Microscopy

The surface morphology and the elemental composition of the AAO membranes prepared using different electrolytes and at different anodisation times were examined using a scanning electron microscope (SEM) coupled with energy dispersive X-rays (EDXs) (JXA-840A, Electron Probe Micro-Analyzer, JEOL, Tokyo, Japan) at 15 kV. They were also examined before and after the drug loading, as well as after annealing at different temperatures. Carbon tape was used to secure the membranes to the stainless-steel sample holders, and all of the samples were Au-sputtered. Structural features such as the pore diameter, interpore distance, pore density, and layer thickness were determined using Equations (1)–(4) after curing the data analysed from the SEM images by the ImageJ software (1.46r). The porosity (δ) was calculated from Equation (1) as follows:

(1) $$\delta =\frac{\pi }{2\sqrt{3}}\times {\left(\frac{D_{p}}{D_{c}}\right)}^{2}=0.907\times {\left(\frac{D_{p}}{D_{c}}\right)}^{2}\;$$

where *D_p_* (in nm) and *D_c_* (in nm) are the AAO pore diameter and the interpore distance, respectively.

Pore density (*p*) is the total number of pores per 1 cm^2^ surface area, and is estimated by Equation (2), as follows:

(2) $$P=\frac{2\times 10^{14}}{\sqrt{3}\times D_{c}^{2}}\;$$

The wall thickness (*W*) can be determined assuming the formation of a typical hexagonal pore from Equation (3), as follows:

(3) $$\begin{array}{l} W=\frac{\left(D_{c}-D_{p}\right)}{2}\; \end{array}$$

The barrier layer thickness (*B*) can be estimated from the wall thickness via Equation (4), as follows:

(4) B=1.12×W

#### 2.3.2. Fourier Transform Infrared Spectroscopy (FTIR)

Before and after the drug loading, the variation in the functional groups on the surfaces of the AAO membranes were examined using FTIR (model FT-IR/ATR, Nicolet 6700 Thermo-Fisher, Norristown, PA, USA) in IR regions from 4000 to 400 cm^−1^.

#### 2.3.3. Contact Angle

One of the major characteristics of implantable drug carriers is their surface wettability, which controls their ability to adhere to the treated site while also facilitating drug release. The contact angle of the AAO membranes was measured using the OCA 15 EC optical contact angle instrument (Data Physics Instrument, Filderstadt, Germany) in accordance with ASTM-D 7334-08 [30]. For the CA measurements, a droplet of distilled water was utilised.

#### 2.3.4. Atomic Force Microscopy (AFM)

The AAO membranes’ three-dimensional topography and roughness were examined using an atomic force microscope (AFM) (XE-100; Park Systems, Suwon, Republic of Korea). To gather the pertinent parameters (Ra), the scanning area of the atomic force probe was set to 25 m^2^ for this test. A WiTec alpha 300 R Raman Imaging Microscope was connected to the AFM. A silicon cantilever with a force constant of 42 N/m and a frequency range of 280–300 kHz was used to record the AFM pictures in non-contractual mode.

### 2.4. In Vitro Drug Loading and Release

Loading was performed using 1 mL of drug dissolved in an artificial cerebrospinal fluid (ACSF) or water (the release of the dissolved drug in water is represented only in Supplementary Materials). The drug was pipetted drop-by-drop onto the membrane surface. Each drop was 100 µL, as the loading step was repeated ten times until the complete loading of 1 mL of the drug solution on each membrane. Between each step, the membrane was left to dry at room temperature. Membranes with dimensions of 1 cm × 1 cm were loaded with two different donepezil concentrations of 10 mg/membrane (named O10 for oxalic AAO membranes and S10 for sulphuric AAO membranes) and 20 mg/membrane (named O20 for oxalic AAO membranes and S20 for sulphuric AAO membranes). After that, the drug release efficiency of the nanoporous membranes was studied for 14 days in ACSF. A volume of 3 mL was withdrawn from each sample and replaced with fresh ACSF at the following time intervals: 1, 3, 5, 24, 72, 120, 168, and 336 h. The drug release profiles were determined by measuring the amount of donepezil released into the ACSF using a UV spectrophotometer at a λ_max_ of 230 nm [31,32].

### 2.5. Donepezil Release Kinetics

The mechanism of the donepezil release in vitro was predicted by comparing the release data with the zero-order, diffusion, and Korsmeyer–Peppas mathematical models [33,34,35] using Equations (5) and (6), as follows:

(5) $$Q=K_{H}t^{\frac{1}{2}}$$

(6) $$\frac{Q_{t}}{Q_{\infty }}=K_{k}t^{n}$$

where *Q_t_*/*Q*_∞_ is the fraction of drug released at time *t*, *K_H_* and *K_K_* are the Higuchi and Korsmeyer–Peppas dissolution rate constants, and n is the kinetic exponent. In the case of quasi-Fickian diffusion, a value of *n* < 0.5; Fickian diffusion = 0.5; for non-Fickian or anomalous transport, *n* = 0.5–1.0; for Case II transport, *n* = 1.0.

### 2.6. Drug Release Efficiency and Kinetics after Heat Treatment

Membranes annealed at different temperatures (500, 600, and 700 °C) were loaded with 20 mg/membrane of the donepezil drug. Drug release was carried out in ACSF for different periods of time, and at the end of the experiment, the drug release kinetics were also studied, as previously mentioned.

## 3. Results and Discussion

### 3.1. Preparation of Alumina Nanoporous Membranes

Figure S1 displays an image of the Al surface after electropolishing in a perchloric acid–ethanol mixture and before the anodisation process. The Al exhibits a flat, shiny, and a mirror-like surface with small pits, which are anticipated to act as seeds for the pore nucleation. Electropolishing typically smooths surface irregularities and creates a high density of small pits that grow into pore nuclei. Moreover, the anodisation of the alumina membranes could be briefly explained as illustrated below:

At first, Al^3+^ ions are formed and diffuse from the surface to the substrate/electrolyte interface (Reaction (7)); then, water electrolysis is carried out, which occurs at the bottom of the pore adjacent to the electrolyte/oxide interface (Reaction (8)). Subsequently, the O^2−^ ions migrate within the barrier layer under the electric field and react with the Al^3+^ ions, forming Al_2_O_3_ (Reaction (9)). The dissolution rate is also continuous (Reaction (10)) [36].

(7) $${Al}_{\left(s\right)}\to {Al}^{3+}+3e^{-}$$

(8) $$2H_{2}O_{\left(aq\right)}\to 2O^{2-}↑+4H^{+}↑$$

(9) $${2Al}_{\left(aq\right)}^{3+}+{3O}^{2-}\to {Al}_{2}O_{3\left(s\right)}$$

(10) $${Al}_{2}O_{3(s)}+{6H}^{+}\to {2Al}_{\left(aq\right)}^{3+}{3H}_{2}O$$

When the porous alumina membranes are anodised, an electric field helps the oxide layer dissolve at the point where the electrolyte and oxide meet. At the interface between the electrolyte and oxide, the electric field speeds up the breakdown of the oxide.

Electrolytes are important because of their protons. It is proposed that, at the bottom of the pores, the protons produced from the dissociation of the acids, besides the water-splitting reaction, are utilised to further dissolve Al_2_O_3_, according to Equation (10). Thus, the dissolution process is an acid-catalysed reaction. In our study, we have focused on two types of electrolytes, namely, 0.3 M oxalic acid (operating voltage (V): 40 V) and 0.3 M H_2_SO_4_ (V = 25 V), to prepare the AAOs with a pore diameter of less than 100 nm through a mild two-step anodisation process.

### 3.2. Detachment of Anodic Film from the Al Substrate

The anodic aluminium oxide (AAO) film was separated from the aluminium substrate by the three methods, mentioned in the Section 2, and the results are summarised in Table 1.

As shown in Table 1 and Figures S2–S4, the through-hole method was successful in piercing the barrier layer and detaching the AAO membrane in the case of the oxalic-acid-prepared film. This method relies on the easy dissolution of the alumina resulting from the anodisation of concentrated H_2_SO_4_. This is because the alumina layer contains sulphate anions. When using sulphuric acid as an electrolyte, the chemical method functions effectively due to its reliance on a single replacement reaction between Al and Cu.

**Figure 2:**
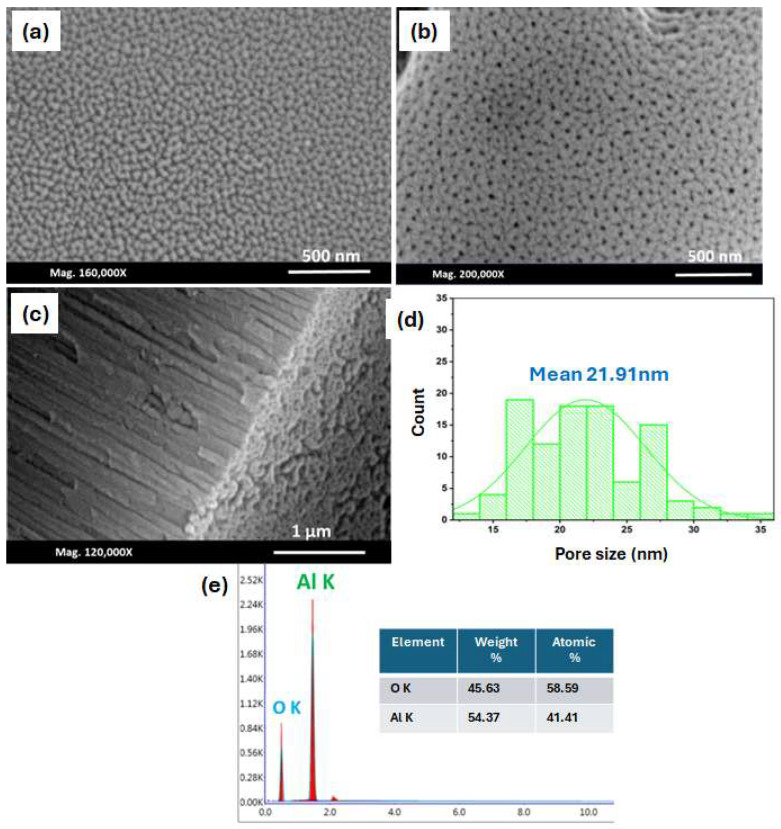
SEM of the Al surface after (**a**) the first anodisation step (5 h) and (**b**) the second anodisation step; (**c**) lateral view of the AAO layer; (**d**) the histogram and (**e**) elemental analysis recorded by the EDX of the AAO after the two-step anodisation process (5 h and 1 h) in 0.3 M oxalic acid at 40 V and a temperature of 0 °C.

We fabricated Al_2_O_3_ via the first anodisation step for 5 h and the second anodisation step for 1 h in 0.3 M oxalic acid, as shown in Figure 2. The micrograph in Figure 2a reveals a high density of pores and parallel grooves. There is minimal separation between the pores, making it difficult to determine their diameter. Pore nucleation may occur during the electropolishing stage in perchloric acid and/or at the beginning of the anodisation in oxalic acid. Figure 2b shows the top-view images after the second ionisation, and, as seen, the pore size increases and the pore density starts to decline. The merging of adjacent pores explains this phenomenon. A hydrogen ion attack on the oxide layer by the generated electric field could explain the pore growth. Therefore, a low hydrogen ion concentration is predicted to cause a slow reaction or the inhibition of the local chemical dissolution at the oxide/electrolyte interface, leading to a pore-free Al surface. Thus, the hydrogen ion attack is essential for pore nucleation and growth, as reported by [36,37,38].

Figure 2c demonstrates the side view of the AAO film, which shows straight cylindrical tubes without any branches or tilting. They are well separated and uniformly aligned tubes with highly controllable pore diameters, confirming the successful formation of the AAO. The average pore volume was determined to be 21.9 nm (Figure 2d). A study of the surface composition of the porous layer was performed with an EDX. Figure 2e shows an atomic ratio of 58.59% for oxygen and 41.41% for aluminium. The chemical composition results prove the formation of a stoichiometric Al_2_O_3_ film. These results were reproducible for all of the fabricated samples at different anodisation times.

### 3.3. Effect of Second Anodisation Time on the Structural Features of Nanoporous Alumina

#### 3.3.1. The Influence of Electrolyte Type (Oxalic Acid)

**Figure 3:**
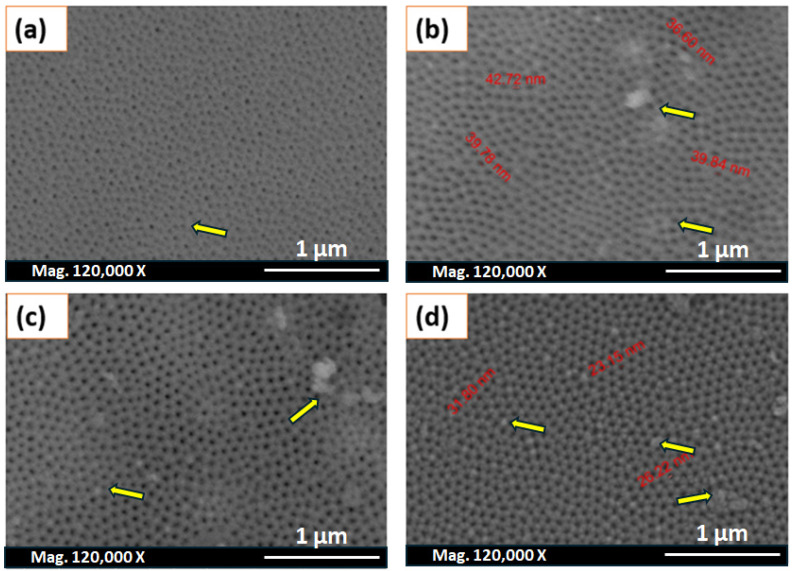
Top-view SEM images of the Al_2_O_3_ membrane surface after the second anodisation for (**a**) 3 h, (**b**) 5 h, (**c**) 7 h, and (**d**) 10 h in 0.3 oxalic acid at 40 V and 0 °C (yellow arrows represents dislocations and grooves found in the samples).

Figure 3 shows the top-view SEM images of different Al_2_O_3_ membranes in 0.3 M oxalic acid performed at constant cell voltage (40 V) and at 0 °C. All of the AAO films were fabricated by first anodising the Al foil for 5 h and a second anodisation step that was performed at different times. The SEM displays similar nanoporous surfaces where the development of point defects and dislocations took place with the increment in the anodisation period. The pore size distribution is also shown in Table 2. When comparing the pore diameter at 1 h (21.91, Figure 2d) to 3 h (38.20, Table 2), it is clear that there is a significant jump. After that, with the variation in the second anodisation time (3–10 h), the average pore diameter (D_P_) was estimated to be around 37 nm in most cases. The anodisation time also did not affect the interpore distance (D_C_) within this range of time (3–10 h). As presented in Table 2, the interpore distance (D_c_) was around 97 nm. Reports indicate that the conversion of Al into Al_2_O_3_ initiates the self-ordering of the pores. The formation of ordered pores, which induce microstructural adjustments and result in hexagonally close-packed arrays, releases compressive stress due to this volume expansion. This expansion is defined by the Pilling–Bedworth Ratio (PBR) [39], which is expressed as follows:

PBR = Volume of oxide produced/Volume of metal consumed(11)

Either the voltage or the electrolyte concentration causes this volume expansion. Volume expansion was explained to result in pushing the oxide in an upward and peripheral direction, thus increasing the length of the pore wall. Thereby, the squeezing in the pore walls is responsible for forming larger pores. There is a linear relationship between the applied voltage and the pore diameter and interpore distance, which is denoted by Equations (12) and (13), as follows:

(12) $$D_{p}=\lambda_{p}\times V$$

(13) $$D_{c}=\lambda_{c}\times V$$

where *λ_p_* and *λ_c_* are the proportionality constants, empirically found to be *λ* ≈ 0.95 nm/V and *λ_c_* ≈ 2.5 nm/V from the literature, and *V* is the applied potential.

In this study, we are using the same electrolyte (0.3 M oxalic acid) and the same voltage (40 V); therefore, based on Equations (12) and (13), the predicted values for the pore diameter and interpore distance should be 38 nm and 100 nm, respectively. When extending the anodisation time, these values should not change. These predictions are in a good match with the results achieved experimentally. The wall thickness and barrier layer thickness show a similar trend to the pore diameter. These findings indicate that no microstructural changes occur in the pores as the time of second anodisation increases. Thus, it is confirmed that the influence of increasing the applied voltage is more significant than anodisation time on controlling the surface characteristics [40]. The decrease in the barrier layer is anticipated to take place in the first anodisation duration owing to the generation of individual pathways through the barrier layer as the porous alumina film is formed under controlled voltage. When the porous alumina membranes are anodised, an electric field helps the oxide layer dissolve at the point where the electrolyte and oxide meet. At the oxide/metal interface, there is a balance between the electric-field-enhanced oxide dissolution and oxide formation [41]. This balance is crucial for the formation of porous alumina membranes, allowing steady-state pore propagation into the Al foil. Bai et al. prepared an AAO film in a mixed acid solution, and they reported that the first anodisation step in H_2_SO_4_ accounted for the changes in the pore diameter [42].

The AAO tubular membrane thickness for the 5 h/10 sample was 145.5 mm, as shown in Figure S5. Belwalkar et al. [43] have reported a membrane thickness of 76 m for an AAO prepared in 20 wt% sulphuric acid under 12.5 V [43]. Yoshimoto and his coworkers conducted anodisation in H_2_SO_4_ for 10 h to prepare an AAO at room temperature, and they attained a maximum film thickness of 100 mm [44]. The increment in the membrane thickness in our study could be attributed to the higher applied voltage versus the reported studies [43,44].

Membrane thickness significantly influences the mechanical strength of the AAO tubular membranes and their resistance to fracture during the fabrication process. Under similar conditions to our study, the growth rate of porous AAO films was found to be 60 nm/min. Based on these studies, we anticipated that our film thickness for different anodisation times to be within 3.6–90 µm for periods from 1 h up to 25 h. However, reports indicate that a prolonged second anodisation time yields a limiting thickness. The dynamic equilibrium between the Al oxidation and the oxide film’s chemical dissolution forms the film. Thus, the oxide formation relies on the hydrogen and oxygen concentrations, and the dissolution process is dependent on the applied field. The AAO’s dissolution rate is believed to limit the membrane thickness and negatively influence the membrane nanohardness.

**Figure 4:**
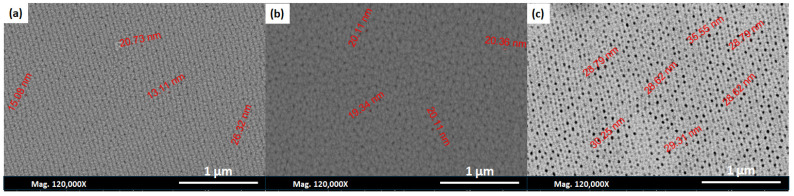
Top-view SEM images of the AAO films that formed in a 0.3 M oxalic acid solution at 0 °C under 40 V anodising voltages after the first anodisation step that took place for (**a**) 50 min, (**b**) 5 h, and (**c**) 9 h, respectively.

It is noteworthy to emphasise here that the slight change in the pore diameter as the second anodisation time increases could be attributed to the formation of pore precursors during the initial anodisation process. It is obvious that the pore diameter increases with time in the first anodisation (as shown in Figure 4a–c). Chahrour and his coworkers [45] have proven that the second anodisation limits the growth of the reconstructed pores, thereby enhancing their depth and increasing the AAO thickness.

**Figure 5:**
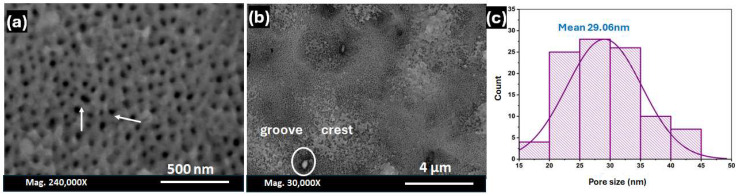
(**a**,**b**) High- and low-magnification top-view SEM of the Al surface after the second anodisation step for 25 h in 0.3 M oxalic acid at 40 V and 0 °C, and (**c**) a histogram depicting the pore diameter.

In order to test the assumption that the second anodisation step has a limited influence on the pore diameter, a longer anodisation time around 25 h was carried out, as shown in Figure 5 for a sheet of Al which was initially anodised at 5 h. The SEM images displayed domains and pore merging, as depicted by the arrows along the domain boundaries. In addition, the SEM micrographs at a low magnification (Figure 5b) demonstrate a superposition of grooves that cover the cylindrical pore structure with crests. When compared to the Al sheets that underwent a short-term second anodisation, the histogram in Figure 5c demonstrates that the mean pore diameter is lowered to 29.06 nm (Table 2). This information leads us to believe that, beyond a certain threshold, the second anodisation period will have a pronounced effect on the pore features.

#### 3.3.2. The Influence of Electrolyte Type (Sulphuric Acid)

**Figure 6:**
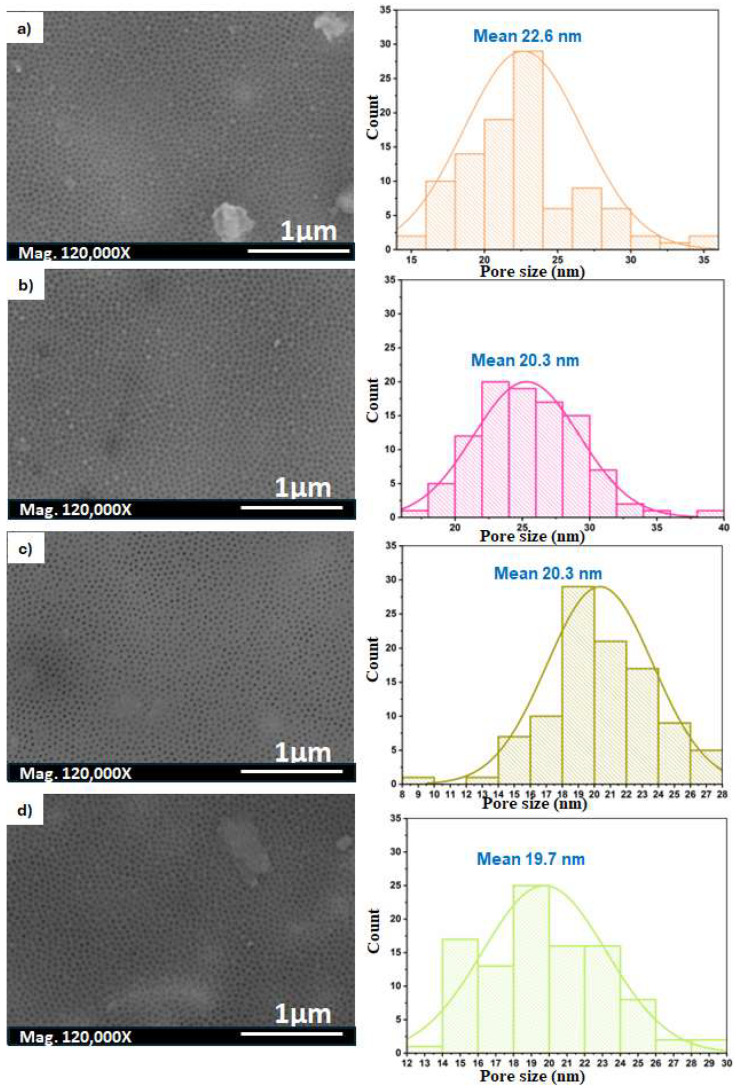
SEM images of the Al_2_O_3_ membrane surface after the second anodisation in 0.3 M H_2_SO_4_ at 25 V and 0 °C for (**a**) 3 h, (**b**), 5 h, (**c**) 7 h, and (**d**) 10 h, and the corresponding images depicting the histograms of the pore diameter for each run.

Anodising Al foil in H_2_SO_4_ solutions forms a porous AAO film, as shown in Figure 6. The films have a narrow range of pore diameters of 19–22 nm, with an interpore distance of approximately 60 nm, as shown in Table 3. These data are consistent with the early reported values [46]. It is noticeable that the films anodised in H_2_SO_4_ have a smaller cell diameter than oxalic acid. The applied voltage could explain this behaviour. The anodisation voltage in the former acid was carried out at 40 V, while in the sulphuric acid it was performed at 25 V. This demonstrates the effect of the increasing potential in providing porous oxide films with larger cell diameters. Additionally, the electrolyte conjugate base plays a critical role in achieving uniform pores [40]. Again, it was observed that the time of the second anodisation step has a slight effect on the pore features. A minor reduction in the pore diameter takes place with increasing the anodisation time from 3 to 10 h at a fixed voltage.

**Figure 7:**
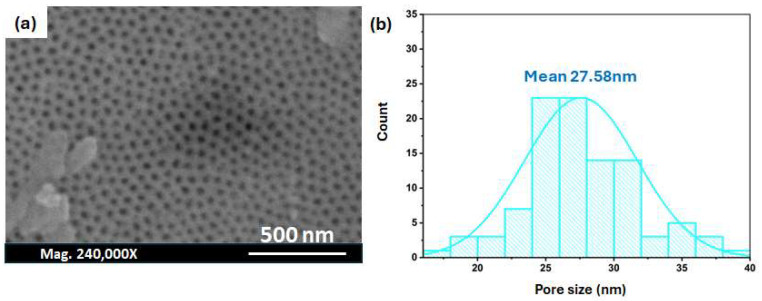
(**a**) SEM of the Al surface after the second anodisation step in 0.3 M H_2_SO_4_ at 40 V and 0 °C, and (**b**) a histogram depicting the pore diameter distribution.

The inspection of the effect of the time of the first anodisation process on the pore characteristics of the membrane prepared in sulphuric acid was tested at a constant voltage, i.e., 25 V. Figure 7 shows the effect of a long anodisation time in both steps (first anodisation = 8 h; second anodisation = 18 h) on the properties of the obtained pores. According to the pore distribution curve, the average pore diameter is 27.58 nm and the interpore distance is 74.9 nm. This experimental observation further supports our hypothesis that the second anodisation process slightly controls the pore properties of the AAO membrane up to a certain value, above which the pore features start to change.

To assess the possibility of using the nano-engineered implants for the targeted delivery of donepezil hydrochloride to brain sites, we have chosen the AAO membrane that was fabricated in both oxalic acid and H_2_SO_4_ for 5 h/10 h and studied its release effects.

#### 3.3.3. Effect of Calcination Temperature on the Pore Features

**Figure 8:**
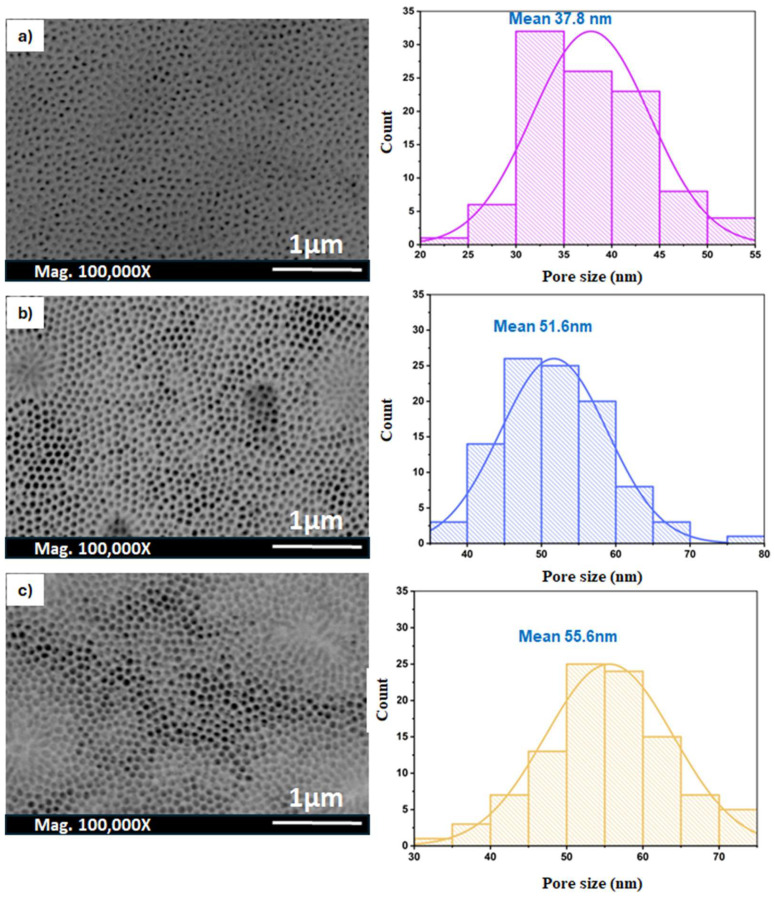
SEM images of the AAO membranes annealed at (**a**) 500 °C, (**b**) 600 °C, and (**c**) 700 °C, respectively, for 3 h, and their corresponding pore size distribution histograms.

Figure 8 illustrates the impact of annealing an AAO membrane on the porous structure of AAO membranes prepared using oxalic acid. Samples annealed at 500 °C showed the smallest pore size, which is approximately 37.8 nm, with no change in the pore features compared to the untreated membranes (Figure 8a). By increasing the annealing temperature from 600 °C to 700 °C, the pores appear to have been widened, as they increased from 51.6 nm to 55.6 nm (Figure 8b,c). Conversely, the thickness of the walls and barrier layers decreased with the increasing temperature, as shown in Table 4. Furthermore, the shape of the pores is hexagonal and more uniform in the samples annealed at 600 and 700 °C.

### 3.4. Surface Morphology of AAO after Drug Loading

**Figure 9:**
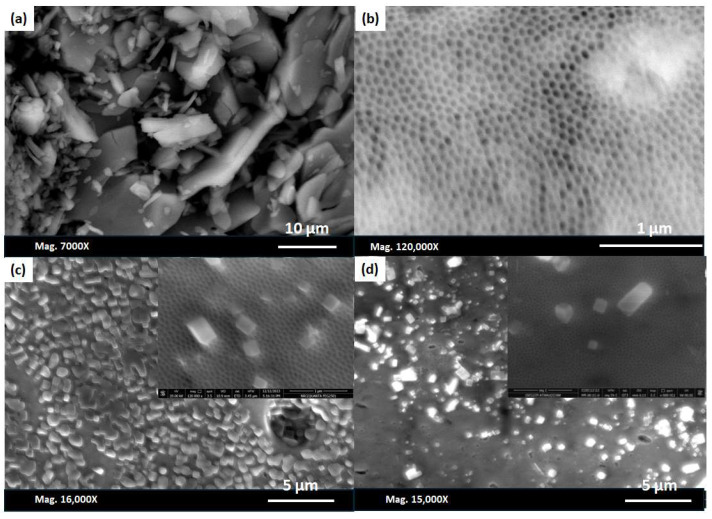
SEM images of (**a**) the drug particles, (**b**) the drug dissolved in water and loaded in the 10 h oxalic-acid-prepared AAO membrane, (**c**,**d**) and the drug dissolved in the ACSF and loaded in the 10 h oxalic- and sulphuric-acid-prepared membranes, respectively.

Donepezil hydrochloride is a highly solubilised drug in both water and ACSF solutions. Figure 9a shows an SEM image of the raw drug, which exhibits platelet shapes of different sizes. Figure 9b reveals the membrane surface after loading the drug solution (dissolved in deionised water) onto its surface. Neither the agglomeration of the drugs on the membrane surface nor the raw drug substance was visible, suggesting that the raw drug either penetrated the membrane pores or adhered to the surface. However, in the case of the drug dissolved in the ACSF, traces of the ACSF were left untreated on the surface of both membranes (prepared either by oxalic acid or H_2_SO_4_), and a random distribution of it was spread on the AAO surface (Figure 9c,d). However, this cannot exclude the possibility of drug loading into the AAO channels. The EDX in Figure S6 shows the presence of Cl and N on the membrane surface, which further confirms the existence of the drug residue on the AAO surface.

### 3.5. FTIR before and after Drug Loading

**Figure 10:**
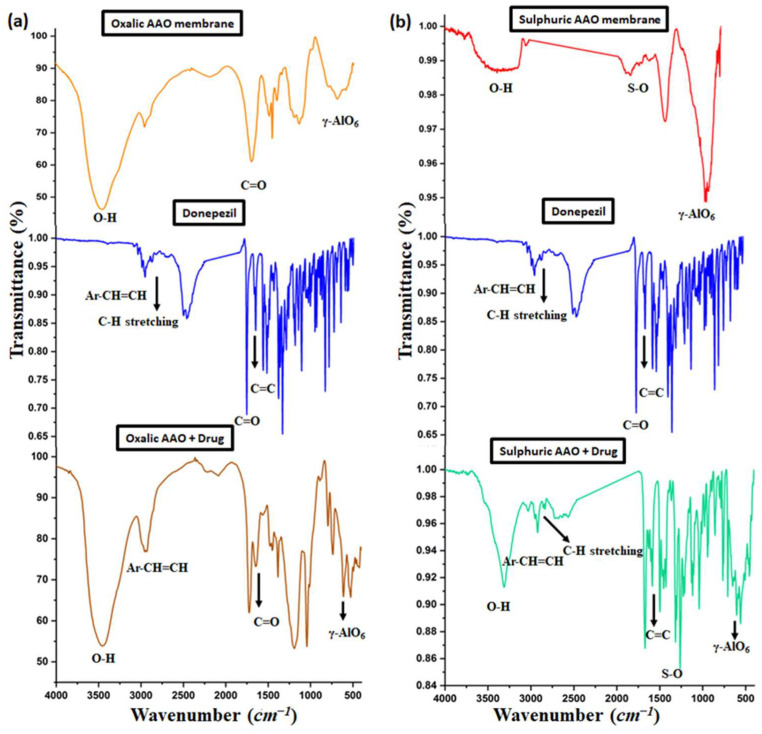
FTIR results of as-prepared AAO membranes in different electrolytes: (**a**) oxalic acid and (**b**) sulphuric acid before and after the drug loading.

The FT-IR spectra were examined to verify the advancement of the donepezil HCl loading into the Al_2_O_3_ channel. The FTIR spectra of the as-prepared AAO membranes fabricated in two different acids before and after drug loading are shown in Figure 10a,b. For the oxalic-acid-prepared AAO membranes (Figure 10a), the results show a broad band at 3437.4 cm^−1^, which is related to the O-H group that appears as a result of adsorbed water from the surroundings. Also, bands that appear at 1634.7 and 600.5 cm^−1^ are assigned to C=O stretching and γ-AlO_6_, respectively [47]. The FTIR spectra obtained for the donepezil HCl (Figure 10a,b) also show four characteristic peaks at 2922.75, 2835.6, 1695, and 1587.91 cm^−1^ corresponding to Ar-CH=CH stretching, the CH stretching of CH_3_ and CH_2_, the C=O ketone stretching band, and C=C bond stretching, respectively [48]. As is evident from the FTIR spectra of both the oxalic-acid- and sulphuric-acid-fabricated AAO membranes, both membranes contain the characteristic peaks of the alumina and drug.

### 3.6. Contact Angle

**Figure 11:**
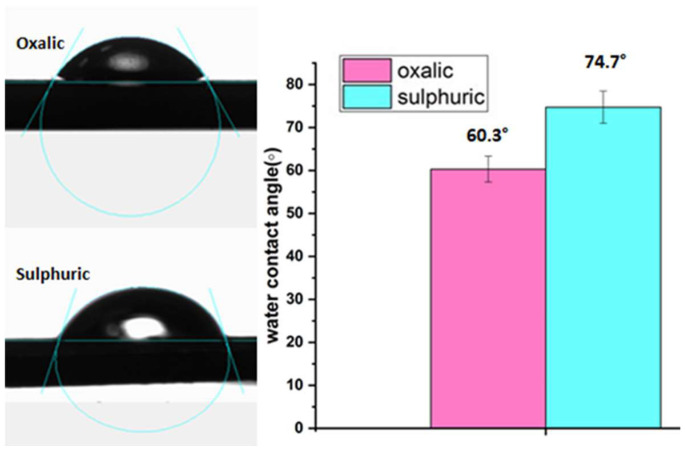
Contact angle of oxalic-acid- and sulphuric-acid-fabricated AAO membranes at 0 °C.

The surface hydrophilicity of the AAO membranes prepared using oxalic acid and sulphuric acid was determined using contact angle measurements. The contact angle is determined by measuring the angle between the surface of the membranes and water droplets. The contact angle instrument tracks the droplet behaviour on the surface until it stops changing. As shown in Figure 11, the results indicate both the oxalic- and sulphuric-acid-prepared membranes exhibit hydrophilic properties. Also, the results have shown that the contact angle of the oxalic-acid-prepared membranes is 60.3°, which is smaller than the contact angle of the sulphuric-acid-prepared membranes, which is 74.7°. The contact angles with the water were highly influenced by the chemical composition of the membranes. This indicates that the oxalic-acid-prepared membranes are more hydrophilic than the sulphuric-acid-prepared ones. The difference in the functional groups present on the membranes’ surface may account for this difference. The presence of COOH functional moieties on the oxalic-acid-prepared membranes makes them more hydrophilic than the S-O-containing membranes [49,50].

### 3.7. AFM of AAO before and after Drug Loading

**Figure 12:**
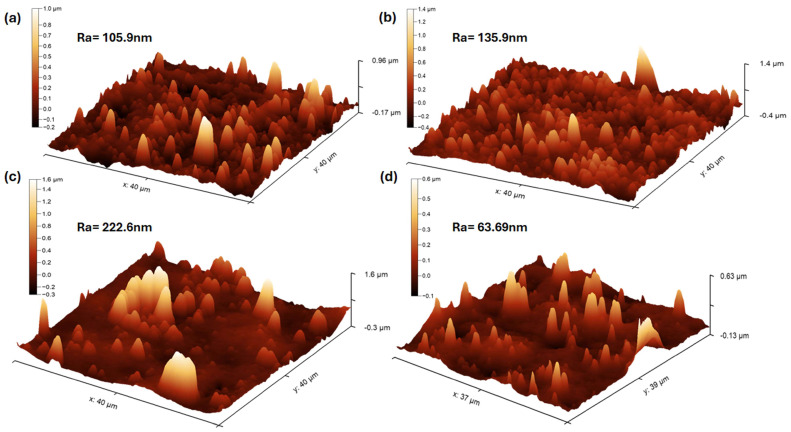
AFM images of (**a**) the oxalic-acid-prepared AAO membrane, (**b**) oxalic-acid-loaded AAO membrane, (**c**) sulphuric-acid-prepared AAO membrane, and (**d**) sulphuric-acid-loaded membrane.

The three-dimensional AFM images for the AAOs of different membranes fabricated in different electrolytes at 5 h/10 h are shown in Figure 12. The AFM analysis demonstrates an increase in the surface roughness of the AAO after drug loading in the case of oxalic acid (Figure 12a,b), resulting in a higher surface area. On the other hand, after drug loading, the surface roughness of the membranes fabricated in H_2_SO_4_ decreased from 222 nm to 63 nm. A more refined topography was observed for the surface of the membrane prepared by oxalic acid as compared to sulphuric acid.

### 3.8. In Vitro Drug Release

**Figure 13:**
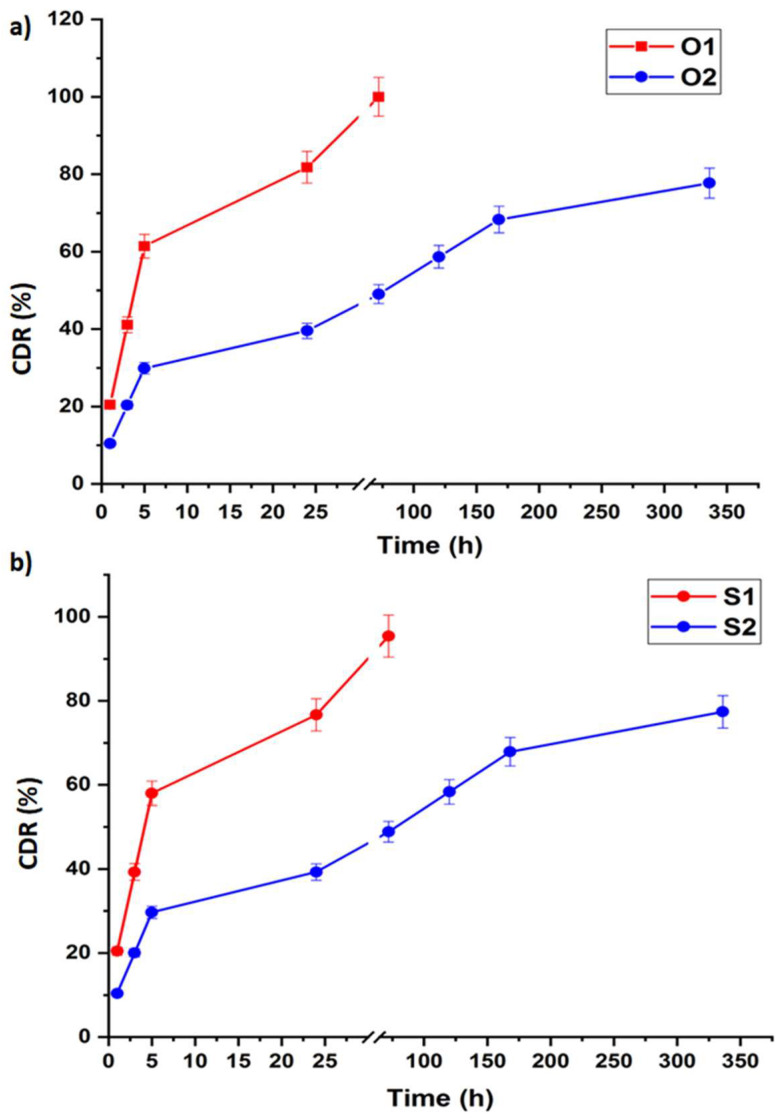
Cumulative donepezil release percentage in the ACSF for 14 days of (**a**) the AAO membranes prepared using oxalic acid and (**b**) the AAO membranes prepared using sulphuric acid.

All of the samples (Figure 13a,b) showed a burst release of donepezil within the first 5 h. This is because the drug is near the surface and is being absorbed or trapped there, making it easy for it to move through the ACSF. The O1 and S1 samples released nearly twice as much drug as the samples with higher concentrations. This could be due to the drug completely filling the pores, slowing down the release from the O2 and S2 samples. The O1 and S1 samples demonstrated a fast and gradual drug release in the following hours (from 5 to 24 h), releasing all of the loaded drug after 3 days from the experiment’s start. On the other hand, during the first hours of the experiment (from 1 to 5 h), the amount of drug released from the O2 and S2 samples was smaller than that of the O1 and S1 samples. Later hours witnessed a gradual and sustained release of the drug. Consequently, the drug released nearly 77.72% of its total amount in the O2 samples and 77.39% in the S2 samples after 14 days. So, as observed, there is no significant difference between the behaviour of oxalic-acid-loaded membranes and the sulphuric-acid-loaded membranes. These results align with the previous literature demonstrating that drug release remains independent of the pore diameter but not of the pore depth, a factor not explored in our research paper [51].

### 3.9. Drug Release Kinetics

In vitro donepezil release from alumina nanoporous membranes was fitted using different mathematical models and the kinetic parameters are listed in Table 5. According to the mathematical calculations, the regression coefficients were 0.889 and 0.95 in the Korsmeyer–Peppas model and 0.86 and 0.94 in the Higuchi model for the O1 and O2 samples, respectively. Also, for the AAO membranes prepared using sulphuric acid, the regression coefficients were 0.90 and 0.948, and in the Korsmeyer–Peppas model and Higuchi model, they were 0.87 and 0.945 for the S1 and S2 samples, respectively. In the present investigation, the Korsmeyer–Peppas model was determined to be the most suitable model for describing the release kinetics of donepezil from the alumina nanoporous membranes. The Korsmeyer–Peppas model is a prevalent mathematical model employed to analyse the release of drugs from porous materials. The release exponent (n) in the Korsmeyer–Peppas model is a vital parameter that defines the release process. The variable n offers valuable data into the prevailing drug release. The release exponent (n) values for the alumina nanoporous membranes loaded with donepezil were documented as follows: 0.35 for O1, 0.31 for O2, 0.34 for S1, and 0.27 for S2. These numbers suggest that the drug release mechanism is mostly characterised by non-Fickian or anomalous transport. These findings indicate that the liberation of donepezil from the nanoporous membranes is affected by processes other than only diffusion, such as membrane swelling, erosion, or the relaxing of the membrane matrix [34,35].

### 3.10. Effect of Temperature on the Drug Release and Kinetics

**Figure 14:**
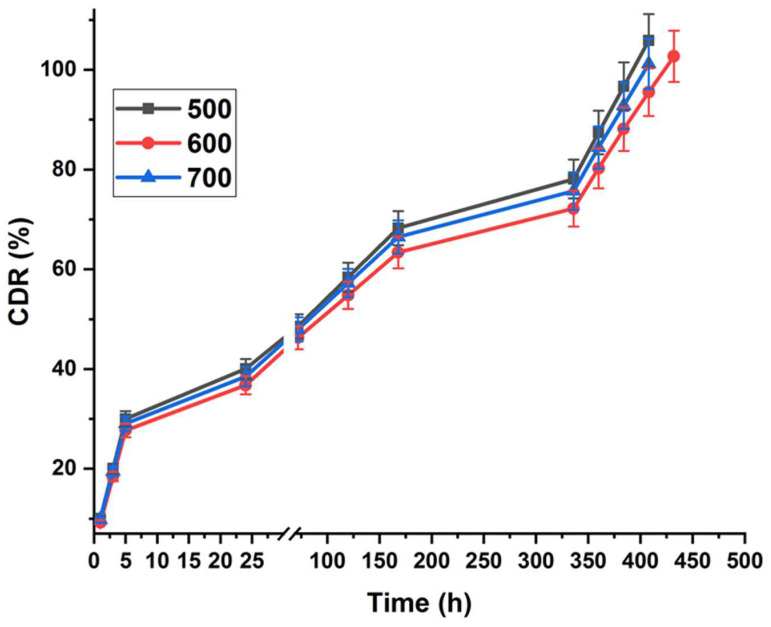
Cumulative donepezil release percentage in the ACSF for 18 days of the AAO membranes prepared using oxalic acid annealed at different temperatures.

Calcined samples at different temperatures were studied for in vitro drug release to evaluate the effect of heat treatment on the drug release and kinetics from the membranes (Figure 14). In the first few hours (from 1 to 5 h), the initial burst release was nearly identical in all of the samples. After that, a slight difference between the samples appeared, as the samples calcined at 600 °C showed a slower release percentage than the other samples. A gradual increase in the drug release was observed from day 1 to day 7. As a result, about 78.11%, 72.18%, and 75.68% of the total amount of the drug were released from the 500 °C, 600 °C, and 700 °C samples, respectively. The release of the samples at 600 °C and 700 °C was slightly less than the release of the untreated membranes; therefore, the experiment was extended a few days in order to reach the maximum release. Samples annealed at 500 °C and 700 °C reached 100% after 17 days, whereas samples annealed at 600 °C reached 100% after 18 days. These results indicated that calcination slowed down the drug release slightly.

From Table 6, it was found that the Higuchi model provided the most accurate representation of the release kinetics of donepezil from the calcined membranes. The Higuchi model is a commonly employed mathematical model for drug release from matrix systems, in which the rate of release is directly related to the square root of time [52]. This implies that the primary factor controlling the release of the medicine is the process of diffusion across the nanoporous membranes. The R^2^ values quantify the degree of agreement between the Higuchi model and the experimental data, indicating the quality of the fit. A higher R^2^ value, which is closer to one, indicates a stronger correlation between the model and the data.

The Higuchi model constants for the temperatures of 500 °C, 600 °C, and 700 °C are consistently stated as 0.97. These constants indicate that the rate at which donepezil is released from the AAO membranes is relatively constant. The t_90_ values, which indicate the duration needed for 90% drug release, are documented as 357.34, 404.33, and 378.92 h for each corresponding case. The Higuchi model offers valuable insights into the release mechanisms and kinetics of donepezil from the calcined alumina nanoporous membranes. According to the model, drug release is primarily controlled by diffusion, as indicated by the relationship between the square root of time and the drug release rate. The Higuchi model proposes that the diffusion process is the main factor controlling the release of donepezil from the nanoporous membranes. In this scenario, the drug molecules permeate the nanoporous structure of the membranes, eventually discharging into the surrounding medium as time progresses [53]. It is suggested that the temperature affects the behaviour of the nonporous membranes. The release mechanism of donepezil from the calcined nanoporous membranes can be explained by the interaction of various factors, such as the size and structure of the pores in the membranes, the solubility of the drug, the interactions between the drug and the membranes, and the difference in concentration between the drug-loaded membranes and the surrounding medium [54]. However, it is worth mentioning that other factors, such as swelling, erosion, or the relaxation of the membrane matrix, may also play a role in the overall release behaviour. These further mechanisms, while not directly accounted for in the Higuchi model, could impact the rate at which donepezil is released from the calcined nanoporous membranes.

## 4. Conclusions

This study effectively improved and created AAO nanoporous membranes by using a two-step electrochemical anodisation procedure, employing various electrolytes and anodisation durations. By manipulating the anodisation duration and the electrolyte type, we were able to accurately regulate the pore diameter. Upon comparing the two electrolytes under examination, it was discovered that the pores created in sulphuric acid were smaller in size and had shorter distances between them compared to the pores formed in oxalic acid. In addition, the consistency and form of the pores were more evident in oxalic acid. Moreover, when anodised for the same duration, the pore density was greater in oxalic acid compared to sulphuric acid, leading to a higher ratio of surface area to volume. This attribute renders the membranes highly suitable for medication delivery applications. We have verified the effectiveness of these nanoporous membranes for continuous medication release by conducting tests on the drug release behaviour. The diffusion control mechanisms were identified to influence the release kinetics of donepezil, which is a model drug. The data suggest that the liberation of donepezil from the membranes predominantly takes place via the process of drug molecules diffusing through the nanoporous structure. Our research indicates that the produced nanoporous alumina membranes have favourable characteristics that make them suitable for use as drug carriers in a variety of drug delivery applications. The ability to precisely manipulate the size, the uniformity, and the high ratio of the surface area to volume makes them highly attractive contenders for effective and regulated drug delivery systems.

## Figures and Tables

**Table 1:** The different separation methods used and the obtained results.

	Separation	Chemical Method	Voltage Pulse Detachment	Through-Hole Method
Samples				
Oxalic-acid-prepared AAO (O)		Failed; membrane cracking and a very slow reaction	Failed; very fine AAO fragments in the solution	Succeeded; a free-standing AAO membrane was obtained
Sulphuric-acid-prepared AAO (S)		Succeeded, but a very slow reaction	Failed; very fine AAO fragments in the solution	Failed; cracked AAO membrane

**Table 2:** Characteristics of the AAO prepared at a fixed potential (40 V), electrolyte concentration (0.3 M oxalic acid), temperature (0 °C), and at different second anodisation times.

Sample ID (First/Second Anodisation)	Pore Diameter D_p_ (nm)	Interpore Distance D_c_ (nm)	Porosity (%)	Pore Density (Pore/cm^2^)	Wall Thickness (nm)	Barrier Layer Thickness (nm)
5/3	38.20	97.40	14	1.22 × 10^10^	78.3	87.69
5/5	39.60	99.37	14	1.17 × 10^10^	79.57	89.12
5/7	37.03	98.81	13	1.18 × 10^10^	80.29	89.93
5/10	32.53	92.43	11	1.35 × 10^10^	76.16	85.30

**Table 3:** Characteristics of the AAO prepared at a fixed potential (25 V), electrolyte concentration (0.3 M sulphuric acid), temperature (0 °C), and at different second anodisation times.

Sample ID (First/Second Anodisation)	D_p_ (nm)	D_c_ (nm)	Porosity (%)	Pore Density (Pore/cm^2^)	Wall Thickness (nm)	Barrier Layer Thickness (nm)
5/3	22.6	58.9	13	3.33 × 10^10^	47.6	53.31
5/5	20.3	59.9	10	3.22 × 10^10^	49.75	55.72
5/7	20.3	60.4	10	3.17 × 10^10^	50.25	56.28
5/10	19.7	63.3	8	2.88 × 10^10^	53.45	59.86

**Table 4:** Characteristics of the AAO membranes at different annealing temperatures.

Heat Temperature of Oxalic Membranes	Pore Diameter D_p_ (nm)	Interpore Distance D_c_ (nm)	Porosity (%)	Pore Density (pore/cm^2^)	Wall Thickness (nm)	Barrier Layer Thickness (nm)
500	37.8	92	15	1.36 × 10^10^	73.1	81.87
600	51.6	95.7	26	1.26 × 10^10^	69.87	78.25
700	55.6	97.24	29	1.22 × 10^10^	69.44	77.77

**Table 5:** Release kinetics parameters of donepezil-loaded alumina nanoporous membranes.

Formula Code	R^2^ Value ^†^			Korsmeyer–Peppas Model		n	Maximum Release %
	Zero-Order Model	Korsmeyer–Peppas Model	Higuchi Model	t_50_ ** (hours)	t_90_ *** (hours)		
O1	0.72	0.889	0.86	4.24	8.62	0.35	100
O2	0.79	0.95	0.94	10.31	19.72	0.31	77.72
S1	0.74	0.90	0.87	4.41	9.03	0.34	95.40
S2	0.79	0.948	0.945	10.37	19.84	0.27	77.39

^†^ R^2^-value is the value of the regression co-efficient, t_50_ ** is time required for 50% of the drug to be released, and t_90_ *** is time required for 90% drug release.

**Table 6:** Release kinetics parameters of donepezil-loaded calcined alumina nanoporous membranes.

Heat Temperature of Oxalic Membranes	R^2^ Value ^†^			Higuchi Model		n	Maximum Release %
	Zero-Order Model	Korsmeyer–Peppas Model	Higuchi Model	t_50_ ** (hours)	t_90_ *** (hours)		
500 °C	0.91	0.96	0.97	78.34	357.34	0.33	100
600 °C	0.92	0.96	0.97	93.30	404.33	0.33	100
700 °C	0.91	0.96	0.97	84.25	378.92	0.33	100

^†^ R^2^-value is the value of the regression co-efficient, t_50_ ** is time required for 50% of the drug to be released, and t_90_ *** is time required for 90% drug release.

## Data Availability

Data are contained within the article and Supplementary Materials.

## Supplementary Materials

The following supporting information are downloaded at: https://www.mdpi.com/article/10.3390/nano14131078/s1, Figure S1: Electropolished Al surface; Figure S2: The dissolution of Al metal in the copper chloride solution; Figure S3: The voltage pulse detachment method; Figure S4: The free-standing AAO membrane obtained by the wet etching of the two-layered anodic porous alumina; Figure S5: SEM image of the cross-section view of the AAO tubular membrane on the Al foil anodised in 0.3 M oxalic acid at 40 V at 0 °C for 5 h/10 h; Figure S6: EDX of drug-loaded Al_2_O_3_ membranes: (a) oxalic-acid-prepared membrane; (b) sulphuric-acid-prepared membrane; (c) drug dissolved in H_2_O; and Figure S7: Dissolution profiles of donepezil HCl dissolved in water and ACSF, respectively.
