# Supplementary material for: Fabrication and Optimisation of Alumina Nanoporous Membranes for Drug Delivery Applications: A Comparative Study
Source: Nanomaterials (Basel). 2024 Jun 24;14(13):1078. doi: 10.3390/nano14131078 (PMC11243695; doi:10.3390/nano14131078)
Supplement: Supplementary file 1 [file nanomaterials-14-01078-s001.zip › nanomaterials-3034458-supplementary.pdf]

Supplementary Information

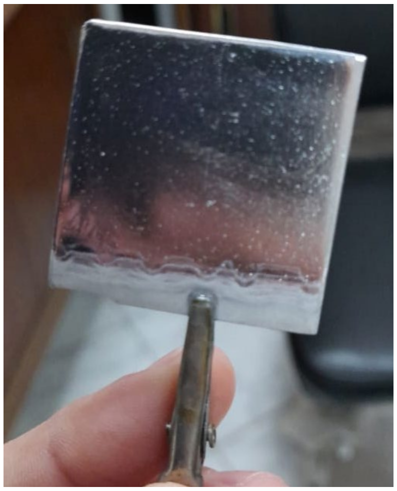

Figure S1. Electropolished Al surface.

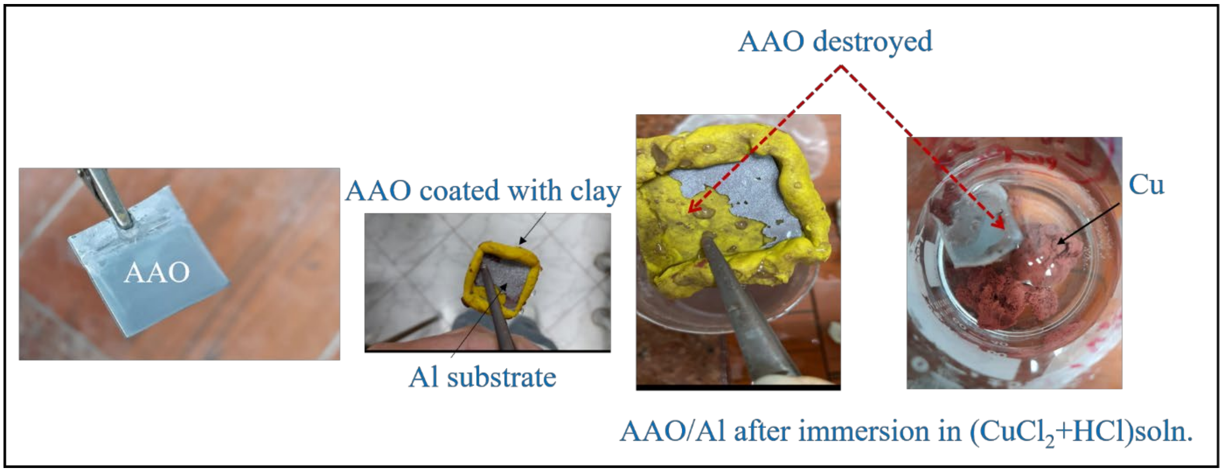

Figure S2. The dissolution of Al metal in cupper chloride solution.

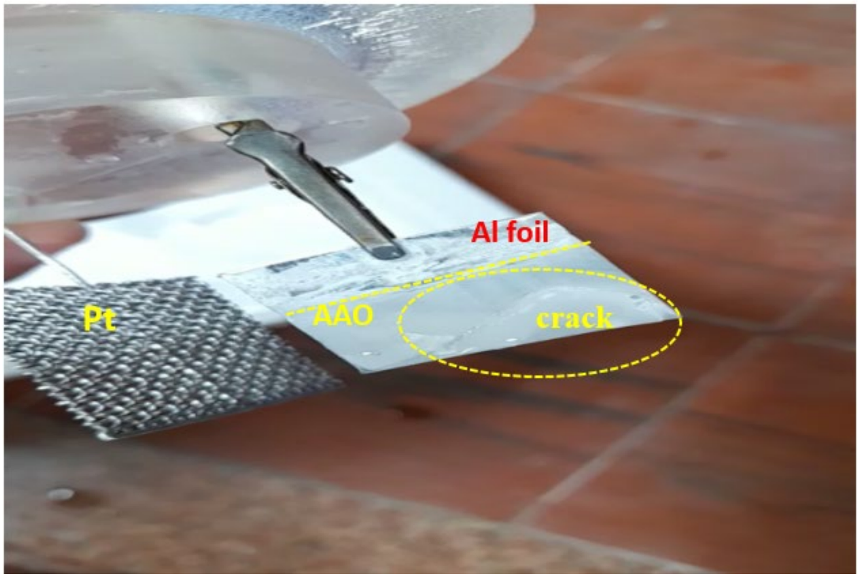

Figure S3. The voltage pulse detachment method

.

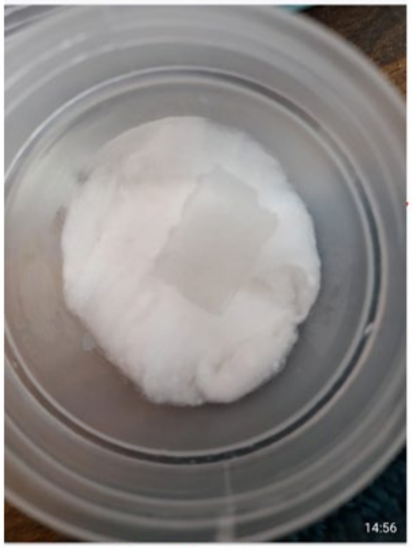

Figure S4. The free standing AAO membrane obtained by wet etching of two-layered anodic porous alumina.

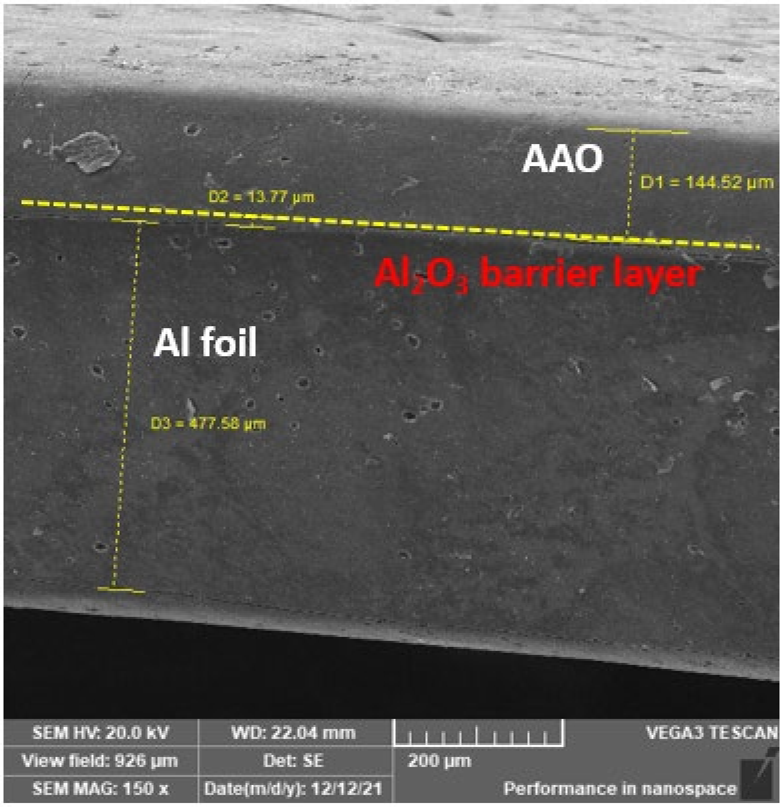

Figure S5. SEM image of cross section view of AAO tubular membrane on Al foil anodized in 0.3 M oxalic acid at 40 V at 0 ^{0}C for 5h/ 10h.

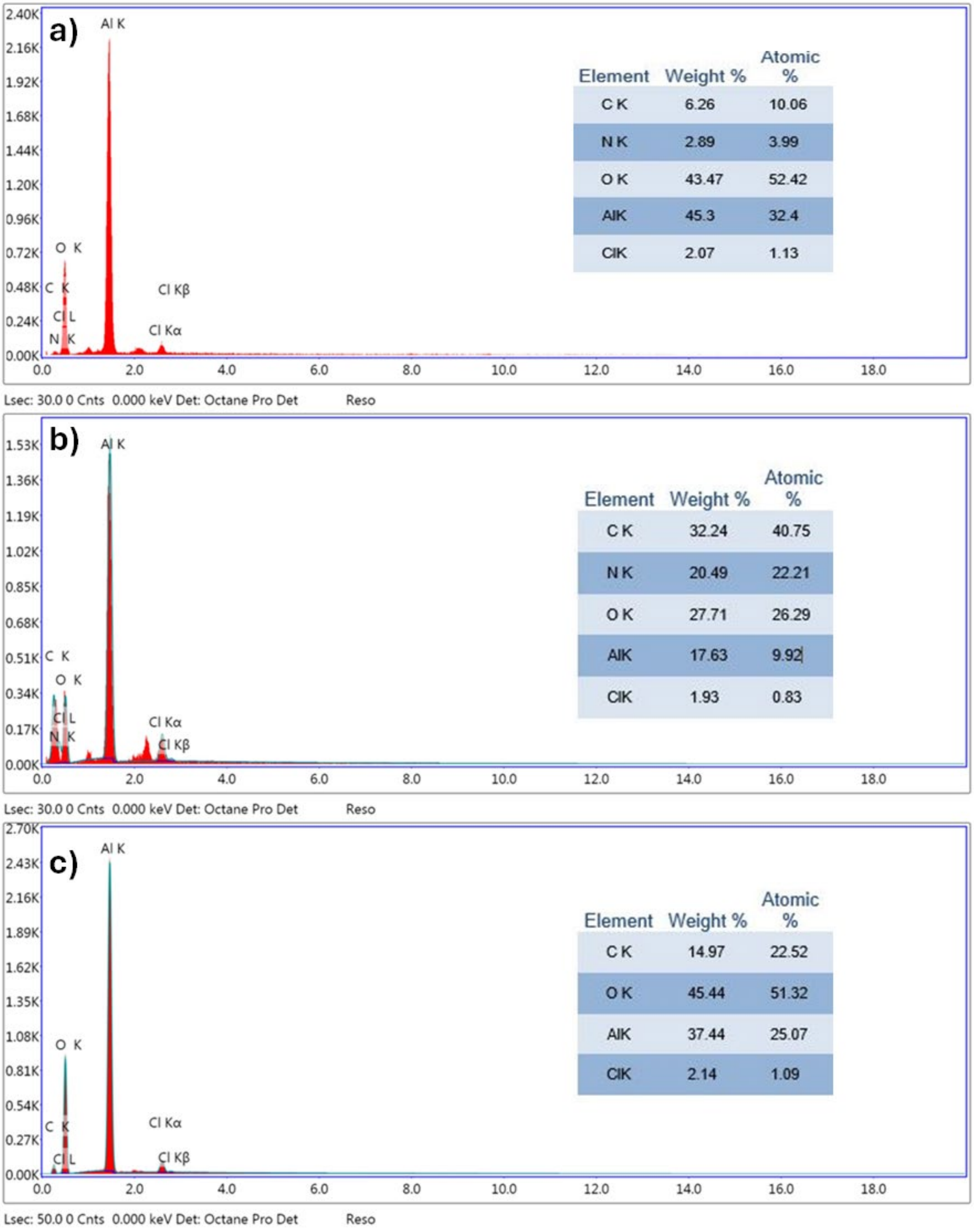

Figure S6. EDX of drug loaded Al_2O_3 membranes a) Oxalic prepared membrane b) sulphuric prepared membrane c) drug dissolved in H_2O.

Drug was dissolved in both water and ACSF and there was no difference in solubilities and drug release from both solutions as shown in figure S7. Therefore, the drug release test was done in ACSF.

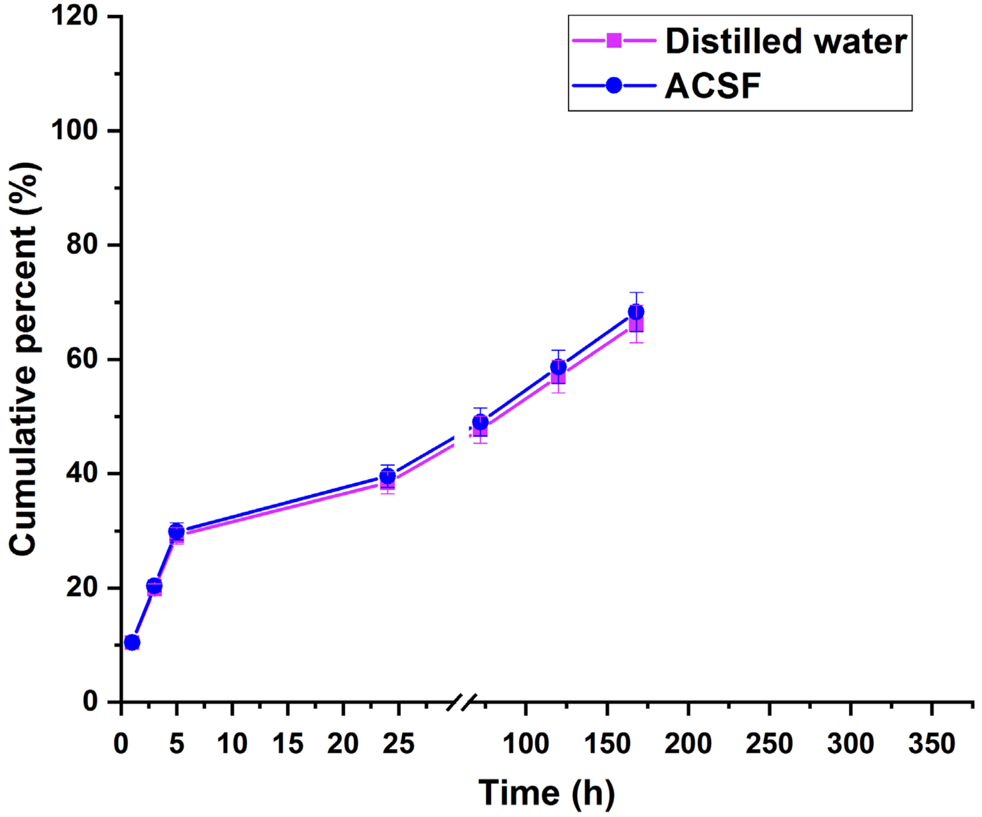

Figure S7. Dissolution profiles of donepezil HCl dissolved in water and ACSF, respectively.
